# Stress and emotion in a locked campus: the moderating effects of resilience and loneliness

**DOI:** 10.3389/fpsyg.2023.1168020

**Published:** 2024-01-08

**Authors:** Qiuwen Wang, Gonggu Yan, Yueqin Hu, Geyi Ding, Yidie Lai

**Affiliations:** School of Psychology, Beijing Normal University, Beijing, China

**Keywords:** COVID-19, school, positive emotion, negative emotion, loneliness, resilience, stress, DSEM

## Abstract

The aim of this study is to investigate the dynamic relationship between Chinese students’ emotions and stress during a strict lockdown period in a university setting and the context of a global pandemic. Dynamic structural equation modeling was used to investigate the moderating role of resilience and loneliness in this relationship. The participants consisted of 112 students. Based on loneliness and resilience measures and the intensive tracking of emotional stress over a 21-day period, the results of data analysis indicated that the students’ overall levels of positive emotions were low and relatively independent of negative emotions. Negative emotions were significantly autoregressive and their baseline was closely related to the individual’s overall feelings of stress and loneliness levels, fluctuating with feelings of stress. The results confirm the hypothesis that resilience helps to stabilize emotions. Individuals with low resilience may be more emotionally sensitive in confined environments, while receiving social support may help to alleviate low moods.

## 1 Introduction

### 1.1 Covid-19, lockdown and emotion

The end of 2019 saw the onset of the novel coronavirus (SARS-COV-2) epidemic which subsequently spread globally and changed people’s lives in many ways (Herbert et al., 2021). To control the scale of transmission, the Chinese government advised all residents, especially school students, to maintain a physical distance from other people (Wang et al., 2020). Confinement restricts individual freedom and conflicts with their actual life and social needs. As the duration of the epidemic and lockdown, the related perception of risk increase, uncertainty and anxiety also increased (Marzana et al., 2022).

In general, isolated and closed environments can have widespread negative psychological effects on the general population (Di Blasi et al., 2021). A series of research studies have explored the impact of social isolation and loneliness during the COVID-19 pandemic on the mental health of different populations. The results indicate that feelings of loneliness have a negative impact on both health and happiness, and can lead to issues such as anxiety, depression, sleep problems, and suicidal tendencies (Czeisler et al., 2020; Loades et al., 2020; Palgi et al., 2020; Duong, 2021).

To control the scale of transmission, the Chinese government advised all residents to maintain a physical distance from other people (Wang et al., 2020). During the COVID-19 pandemic, a large number of colleges and universities locked down their campuses in varying degrees to restrict student access. A review during the epidemic also showed an association between social isolation and an increased risk of mental health problems in young people, including depression, anxiety, and other psychiatric disorders. It was also strongly associated with the duration of loneliness (Loades et al., 2020). approximately 25% of Chinese student participants had increased levels of anxiety during the pandemic (Cao et al., 2020). Studies from other countries have also demonstrated that isolation is associated with negative psychological conditions among college students (Sahu, 2020; Wathelet et al., 2020). There are also studies that show differences in the degree of performance and tolerance of limitations in life among different personalities (Quigley et al., 2022). loneliness do not necessarily correspond with external social isolation, emphasizing the discrepancy between social expectations and social experiences (Labrague et al., 2021). From social cognitive model perspective, individuals with strong feelings of loneliness may be more sensitive to threat and experience deeper levels of stress (Cacioppo and Hawkley, 2009). Compared to people with low levels of loneliness, they may exhibit higher levels of negative impact and lower levels of positive impact (van Roekel et al., 2014). Feelings of loneliness may play a moderating role between the experience of stress and emotional levels (van Roekel et al., 2015).

### 1.2 Resilience

Resilience is defined as the ability to recover from adversity, threat, or trauma (Feder Fred-Torres et al., 2019). Resilience can benefit individuals in two dimensions: on the one hand, it helps resist the negative effects of adversity, and on the other hand, it can enhance individual well-being and promote positive development (Kaye-Kauderer et al., 2021). Stress and setbacks are an integral part of daily life. The COVID-19 pandemic as a global disaster has brought even more challenges to people’s everyday lives, providing an opportunity for us to study and understand resilience (Labrague et al., 2021).

Social support, personal adaptability, and coping abilities are considered protective factors against adversity and stressful conditions such as disasters and disease outbreaks (Turner, 2015; Zhang et al., 2020). Existing research suggests that resilient individuals and those with adequate support systems and coping skills are less likely to feel stressed or lonely in stressful events (Ogińska-Bulik, 2018; Masten et al., 2021). It has also been observed that sufficient support from peers and family is crucial in helping individuals effectively manage situations such as disasters, emergencies, and infectious disease epidemics (Langan et al., 2017). During the COVID-19 pandemic when both stress and loneliness levels are high, personal adaptability, positive coping behaviors, and adequate social support can help individuals cope effectively with the burdens associated with the pandemic and maintain their psychological well-being (Yu et al., 2020).

Some personal traits or talents related to resilience can help individuals interpret ordinary or even negative events in their lives in a positive light and help alleviate stressful experiences. Such traits include motivation (Ghanizadeh et al., 2019), hope (Hinkle Jr, 1974), humor (Bhattacharyya et al., 2019), and “self-determination” (Burtaverde et al., 2021). Additionally, resilience helps individuals to engage in functional and meaningful socialization, which can help individuals to gain a sense of belonging (Habersaat et al., 2020). This has important implications for mitigating the negative effects of isolation under a lockdown situation (Graupensperger et al., 2020; Loades et al., 2020). In addition, Ong et al. (2006) used multilevel contingency modeling to verify that personal resilience can facilitate recovery from negative emotions by increasing an individual’s level of positive emotions.

### 1.3 Dynamic structural equation modeling

With advances in psychological research methods, methods such as experience sampling and ecological momentary assessment enable researchers to collect longitudinal data in a less invasive and intensive manner (Trull and Ebner-Priemer, 2014). Such data patterns also allow longitudinal studies on response development processes to further investigate dynamical processes over a relatively stable timeframe (Molenaar and Campbell, 2009).

Most of the current articles related to students’ emotions and negative psychological conditions in the context of the pandemic use cross-sectional studies and traditional tracing, but fail to draw causal conclusions (Zhang et al., 2020). Such an approach can account for current emotional states and overall changes, but cannot explore the dynamic processes under relatively stable high negative emotions (McNeish and Hamaker, 2020). In this paper, we use an intensive tracking study to measure students’ emotional states during strict campus closures, employ dynamic structural equation modeling to explore the relationship between emotional experiences and stress among college students during the lockdown period, and to examine the moderating roles of resilience and loneliness.

## 2 Materials and methods

### 2.1 Participants

The participants consisted of 112 students who were recruited through alumni groups and campus postings during the winter break lockdown management, 2022. The participants’ age range was 18–32 years old. There were 87 females (78%), 45 undergraduates (40%), 53 masters (47%), and 14 PhDs (13%). The recovered questionnaires were sorted and matched, firstly eliminating duplicate and inattentive questionnaires, and then discarding subjects with missing values greater than 60% (Barzi and Woodward, 2004). A total of 88 questionnaires were obtained as a result, with 63 data points per participant, and 4,978 data points were obtained by excluding missing data, with a valid response rate of 89.8%.

### 2.2 Procedures

Participants signed an informed consent form and voluntarily joined the study cohort. The study questionnaire was compiled on the questionnaire website https://www.wjx.cn/. The positive and negative mood scales and stress questions were pushed via letter three times a day from February 8, 2022 to February 28, 2022. February 8 and February 28 each contained a pre-test and post-test questionnaire containing a loneliness scale and a basic resilience scale in addition to the daily mood questionnaire and stress perception assessment. The daily questionnaires were administered at 10:00 am, 16:00 pm, and 22:00 pm. Participants responded to the questionnaire by logging in with their experiment number.

### 2.3 Materials

#### 2.3.1 UCLA loneliness scale

Twenty items of the UCLA loneliness scale (Russell, 1996) were used to measure loneliness in both the pre-test and post-test. Participants responded on a Likert-type scale of 1 to 4, with 1 indicating “never” and 5 indicating “often.” The scale contains 11 negative (lonely) and 9 positive (non-lonely) items, all of which can be administered easily through a personal interview (Shevlin et al., 2015). The higher the score, the greater the isolation. In the present study, the Cronbach’s alpha coefficient of the scale was 0.927.

#### 2.3.2 Essential resilience scale

Several Chinese researchers have developed an operational definition of resilience, which include the factors of flexibility, anticipation, and “bounce-back.” Based on this, they developed an essential resilience scale (ERS) with high reliability and validity among rural and urban Chinese residents (Chen et al., 2016). The 15-item ERS was used to measure overall global trait resilience in the pre-test and post-test. Participants responded on a scale of 1 to 5, with 1 indicating “strongly disagree” and 5 indicating “strongly agree.” The higher the score, the greater the individual’s psychological resources. In this study, Cronbach’s alpha for the scale was 0.870.

#### 2.3.3 Positive and negative affect scale

The 20 items of the positive and negative affect scale (PANAS) (Clark and Tellegen, 1988) were used to measure daily positive and negative emotions. Participants were asked to indicate the extent to which these emotions were experienced during the day and to respond on a scale of 1 to 5, with 1 being “very mild or not at all” and 5 being “very severe.” The negative activation subscale of the PANAS has 10 items (fear, shame, distress, guilt, hostility, irritability, tension, nervousness, fear, and restlessness) and the positive activation subscale has 10 items (positivity, change, concentration, determination, enthusiasm, excitement, inspiration, interest, pride, and strength). All these items represent a wide range of pleasant and unpleasant emotional states.

#### 2.3.4 Stress questionnaire

Before PANAS, we used a stress questionnaire to get participants’ assessment of stress during the last time interval. Participants were given 10 s to recall a stressful event in the past few hours. Then they responded to a single question on a scale of 1 to 5, with 1 being “very little” and 5 being “very much” (Ong et al., 2006), with the voluntary option to describe the stressful event.

### 2.4 Data analysis

Descriptive and correlation analyses were conducted using IBM SPSS Version 25. Dynamic structural equation models were built using Mplus 8.3, which has a dedicated module for dynamic structural equation modeling (DSEM) for processing intensive longitudinal data (ILD) (Asparouhov, 2018). Intra-individual dynamics can be modeled for time series data while individual differences are probed by individual parameters (Brose et al., 2015; Jebb and Tay, 2017).

To test the moderation hypothesis, we established Equations 1–7. Simulation studies are considered to be effective in dealing with missing values using 50,000 Markov Chain Monte Carlo iterations (Schultzberg and Muthén, 2018). Daily questionnaire distribution was concentrated at 10:00 am, 16:00 pm, and 22:00 pm. Recoding and the statement TINTERVAL = time(1) were used to deal with the problem of unequal time intervals (Hamaker and Wichers, 2017; McNeish and Hamaker, 2020). Symmetrically, positive emotions were operated as dependent variables against negative emotions.


(1) $${\mathrm{NA}}_{ti}=\alpha_{i}+\varphi_{i}{\mathrm{NA}}_{\left(t-1\right)i}^{c}+\beta_{i}\;{\mathrm{STR}}_{ti}^{c}+\eta_{i}{\mathrm{PA}}_{ti}^{c}+e_{ti}$$

(2) $$\begin{array}{l} \alpha_{i}=\gamma_{00}+\gamma_{01}{\mathrm{RES}}_{i}^{c}+\gamma_{02}{\mathrm{LON}}_{i}^{c}+\gamma_{03}\;{\mathrm{STR}}_{i}^{b}\\ \;\;\;\;\;\;\;+\gamma_{04}{\mathrm{PA}}_{i}^{b}+u_{0i} \end{array}$$

(3) $$\varphi_{i}=\gamma_{10}+\gamma_{11}{\mathrm{RES}}_{i}^{c}+\gamma_{12}{\mathrm{LON}}_{i}^{c}+u_{1i}$$

(4) $$\beta_{i}=\gamma_{20}+\gamma_{21}{\mathrm{RES}}_{i}^{c}+\gamma_{22}{\mathrm{LON}}_{i}^{c}+u_{2i}$$

(5) $$\eta_{i}=\gamma_{30}+\gamma_{31}{\mathrm{RES}}_{i}^{c}+\gamma_{32}{\mathrm{LON}}_{i}^{c}+u_{3i}$$

(6) $${\mathrm{STR}}_{ti}^{b}=\gamma_{40}+u_{4i}$$

(7) $${\mathrm{PA}}_{ti}^{b}=\gamma_{50}+u_{5i}$$

Equation 1 addresses individual-level (within) questions exploring autoregressive effects, and other variability variables within individuals (Schuurman et al., 2016). Equations 2–5 use the personality variables resilience trait and loneliness as predictors of individual-specific intercepts and use regression coefficients to analyze the relationship between inter-individual variance variables such as personality and variability variables (Schuurman et al., 2016).

Mplus will default to the latent person-mean for DSEM (Asparouhov, 2018), which is ${\mathrm{STR}}_{ti}^{b}$ and ${\mathrm{PA}}_{i}^{b}$ , while ${\mathrm{STR}}_{ti}^{c}$ and ${\mathrm{PA}}_{ti}^{c}$ are the values after being centered relative to the potential individual means. By putting ${\mathrm{STR}}_{ti}^{b}$ and $\;{\mathrm{PA}}_{i}^{b}$ into Equation 2 as covariates of $\alpha_{i}$ , we can distinguish the dynamic effects and mean effects of positive emotions and stress values. More specifically, we are interested in whether individuals are more likely to experience negative emotions on specific occasions of high stress or whether negative emotions are only affected by average stress values and not by single stress fluctuations. Thus, we have the following Equations 8 and 9:


(8) $${\mathrm{RES}}_{i}^{c}={\mathrm{RE}}_{i}^{n}-\bar{{\mathrm{RE}}_{i}^{c}}\;$$

(9) $${\mathrm{LON}}_{i}^{c}={\mathrm{LON}}_{i}^{n}-\bar{{\mathrm{LON}}_{i}^{c}}$$

The trait variables do not vary over time, and only individual differences exist. The residuals in the equations all obey normal distributions: $e_{\mathrm{ti}}\sim N\;\left(0,\sigma 2\right),u_{i}\sim \mathrm{MVN}\left(\left[\begin{array}{c} 0\\ 0 \end{array}\right],\left[\begin{array}{cc} \tau 00 & \tau 01\\ \tau 10 & \tau 11 \end{array}\right]\right)$ .

## 3 Results

### 3.1 Descriptive statistics and correlation analysis

There were 63 measurement points for negative emotions, positive emotions, and stressful experiences. Resilience and loneliness were treated as trait variables in this study and were relatively stable within individuals (Kalisch et al., 2015), with the mean of the two measurements taken as representative. Resilience pre-test and post-test retest reliabilities were 0.772 (*p* < 0.01) and the loneliness retest reliability was 0.875 (*p* < 0.01). Correlations between all constructs in the study with the means and standard deviations are shown in Table 1.

**Table 1:** Correlation of variables.

	NA	PA	STR	RES	LON	M (SD)_within_
NA	1	−0.10^**^	0.561^**^			2.99 (1.19)
PA	−0.01	1	−0.12^**^			2.50 (0.86)
STR	0.69^**^	−0.14	1			1.97 (0.86)
RES	−0.19	0.24^*^	−0.21	1		
LON	0.31^**^	−0.20	0.22^*^	−0.26^*^	1	
M (SD)_Between_	1.97 (0.67)	2.49 (0.69)	3.00 (0.77)	3.13 (0.60)	2.26 (0.55)	

NA, negative affect; PA, positive affect; STR, stress; RES, resilience; LON, loneliness; bold indicates significant correlation; ^**^p < 0.01, ^*^p < 0.05.

### 3.2 DSEM results

The model estimates are shown in Table 2. The intercept α represents the mean level of negative affect for all individuals and is centered at 0.01 (γ_00_) with significant fluctuating variance around it across individual baselines (τ_00_ = 0.31). According to the latent variable hypothesis, 95% of subjects in the data had a specific individual intercept of −0.01 ± 1.96\ √0 .31 between [−1.08, 1.10]. Predictor variables of α included resilience, loneliness, mean stress level, and average stress level, corresponding to coefficients γ_01_, γ_02_, γ_03_, and γ_04_, respectively. Zero was not within the 95% confidence interval of γ_02_ and γ_03_, the covariate coefficients of α. This indicates an increase of 0.17 points in mean negative affect for each unit increase in individual trait loneliness experience and an increase of 0.81 points in mean negative affect for each 1 point increase in individual mean stress experience.

**Table 2:** Model standardized estimates and their 95% confidence intervals.

Effect	Notation	Negative affect		Positive affect	
		Posterior median	95% CI	Posterior median	95% CI
Intercept (α)	γ_00_	0.01	[−0.12, 0.13]	−0.02	[−0.19, 0.16]
Intercept (φ)	γ_10_	0.32	[0.27, 0.39]	0.34	[0.30, 0.39]
Intercept (β)	γ_20_	0.02	[0.02, 0.04]	−0.01	[0.04, 0.04]
Intercept (η)	γ_30_	0.05	[0.03, 0.07]	−0.12	[−0.18, −0.05]
Intercept (STR)	γ_40_	0.01	[−0.15, 0.14]	0.01	[−0.15, 0.14]
Intercept (P/N)	γ_50_	−0.02	[−0.18, 0.16]	0.01	[−0.16, 0.17]
α on RES	γ_01_	−0.02	[−0.15, 0.12]	0.14	[−0.05, 0.12]
α on LON	γ_02_	0.17	[0.04, 0.29]	−0.15	[−0.33, 0.03]
α on STR	γ_03_	0.81	[0.61, 1.00]	−0.30	[0.68, 1.00]
α on P/N	γ_04_	0.13	[−0.03, 0.29]	−0.30	[−0.68, 0.07]
φ on RES	γ_11_	−0.04	[−0.01, 0.01]	−0.02	[−0.07, 0.03]
φ on LON	γ_12_	0.01	[−0.04, 0.06]	0.01	[−0.04, 0.06]
β on RES	γ_21_	−0.05	[−0.09,. -01]	0.02	[−0.02, 0.07]
β on LON	γ_22_	0.01	[−0.03, 0.05]	0.01	[−0.03, 0.05]
η on RES	γ_31_	−0.01	[−0.07, 0.04]	0.03	[−0.11, 0.04]
η on LON	γ_32_	0.04	[−0.02, 0.09]	0.03	[−0.04, 0.10]
Var. (α)	τ_00_	0.31	[0.23, 0.44]	0.61	[0.45, 0.86]
Var. (φ)	τ_11_	0.04	[0.03, 0.06]	0.03	[0.02, 0.05]
Var. (β)	τ_22_	0.02	[0.02, 0.04]	0.02	[0.01, 0.03]
Var. (η)	τ_33_	0.05	[0.03, 0.07]	0.08	[0.05, 0.11]
Var. (STR)	τ_44_	0.43	[0.32, 0.57]	0.42	[0.32, 0.58]
Var. (P/N)	τ_55_	0.65	[0.49, 0.89]	0.62	[0.47, 0.32]
Res. Var. (N/P)	σ^2^	0.21	[0.20, 0.22]	0.28	[0.26, 0.29]
*R*^2^ within		0.35		0.26	
*R*^2^ between		0.50		0.16	

N, negative affect; P, positive affect; bold means 0 is not in the confidence interval; Var., variance; Res. Var., residual variance.

The mean autoregressive coefficient for negative affect was 0.32, with a small variability of 0.04 (τ_11_), while φ includes the predictor variables resilience and loneliness in the formula, corresponding to coefficients γ_11_ and γ_12_. Similarly, the mean coefficient for stress was 0.02, and for each unit increase in resilience, the effect of stress on negative affect was −0.05.

**Figure 1:**
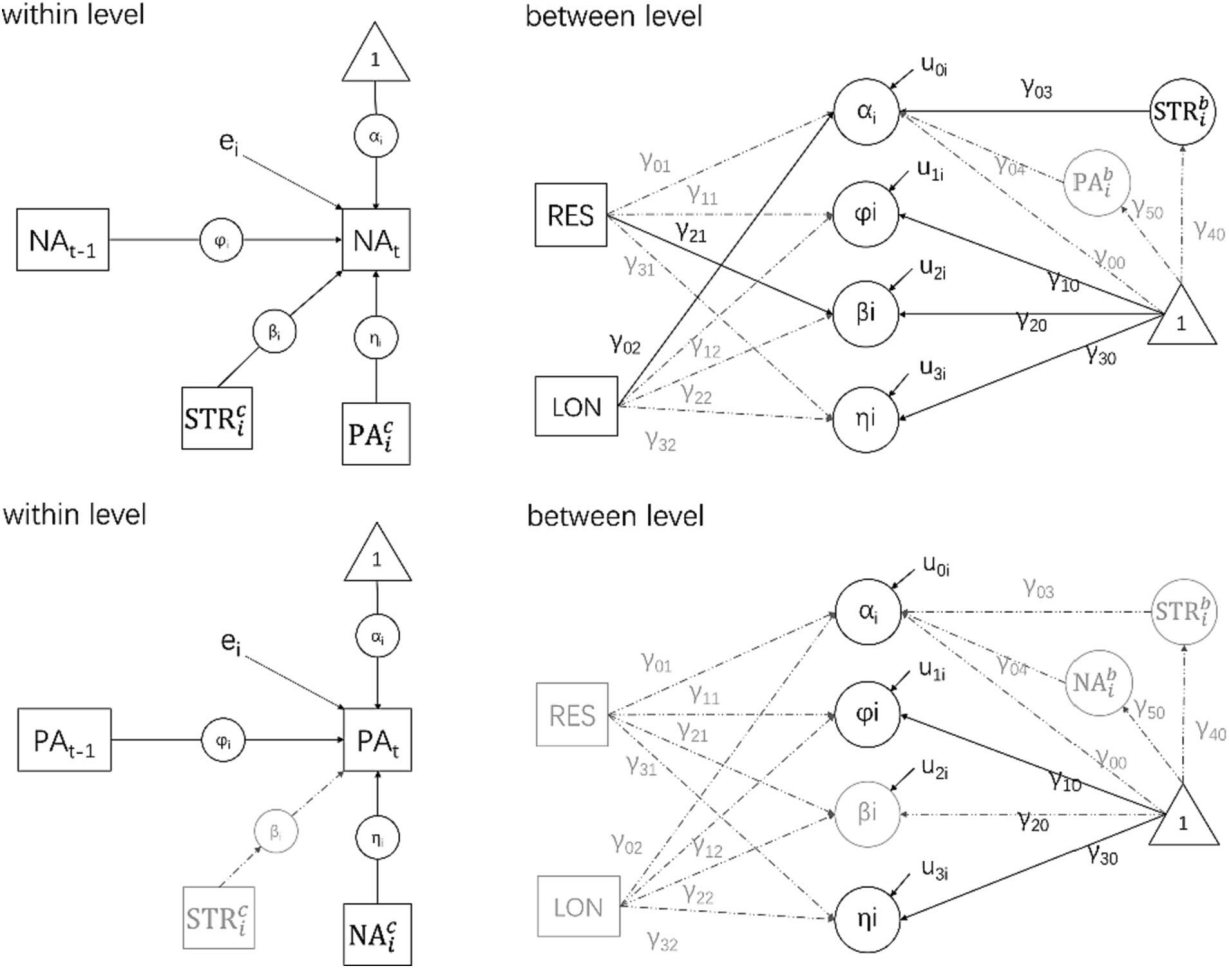
Model path diagram based on Equations 1–7 and the results of the operation.

The mean positive affect across all subjects was 0.02 (γ_00_) with significant individual variance (τ_00_ = 0.61) and a specific individual intercept between [−1.55, 1.51] for 95% of subjects. The autoregressive effect was significant (γ_00_ = 0.34), and fluctuated little (τ_11_ = 0.03). The mean coefficient of negative emotions was −0.12. The model path diagram and simulation results are presented in Figure 1.

## 4 Discussion

Social isolation due to lockdowns is interrelated with loneliness, and this association is prevalent in past global epidemics (Bu et al., 2020; Xiao et al., 2020). The outbreak of COVID-19 resulted in feelings of stress and associated negative emotions that can exacerbate the experience of loneliness (British Red Cross, 2020; Brooks et al., 2020; Holmes et al., 2020). Emotional experiences vary across groups in closure management (Shah et al., 2020). It has been shown that young people, especially students, experienced higher levels of loneliness and were exposed to higher health risks during the COVID-19 pandemic (Bu et al., 2020; Hysing et al., 2020; Pierce et al., 2020). Understanding the relationship between individual feelings of emotional stress and loneliness is important for understanding and helping young people with emotional adjustment (Bu et al., 2020).

Overall, the results of this study suggest that the average level of negative affect among students in a locked campus situation depends heavily on the total average stressful feelings of the individual. It is also constantly fluctuating, influenced by stressful events of the day. The voluntary results completed by some students with incomplete returns indicate that the main source of stress was the academic progress of the day. The data results suggest that this immediate impact is moderated by resilience, and that individuals with high resilience are less likely to have their present-day stress translated into negative emotions, and are more likely to show greater resilience.

Levels of negative emotion were also associated with loneliness, and baseline levels of negative emotion were correspondingly higher in individuals with high feelings of loneliness. Combined with the significant autoregression of negative emotions, the findings confirm that loneliness is associated with negative emotions under the epidemic condition, and that the longer this loneliness is felt, the more difficult it is to dissipate its negative emotional impact cumulatively (Loades et al., 2020).

It is worth noting that positive emotions are not incompatible with negative emotions, and the data suggest that individuals can retain high levels of positive emotions despite high negative emotional states. On the one hand, there is theoretical support for the relative independence of positive and negative emotions (Bergman and Wallace, 1999; Moskowitz et al., 2003; Reich et al., 2003). On the other hand, this may also be related to individual habits of emotional expression, and the data in this study also reflect a notable amount of individual variation in how individuals feel and express negative emotions. In this regard, analysis based on individual means is necessary to avoid overwhelming potential relationships between some variables.

Müller et al. (2021) suggest that emotional problems are adaption problems and that the trait of resilience is important for emotional adaptation, while Ong et al. (2006) suggest that resilience can reduce negative emotions by mobilizing positive emotions. Unlike previous studies, the results of the present study did not show an effect of resilience on the relationship between positive and negative emotions. However, when positive emotions were removed from the equation, the moderating effect of resilience also disappeared. This implies that there may be a more complex relationship between resilience and positive emotions, perhaps understood as higher positive emotions being a component of trait resilience (Tugade et al., 2004; Yi et al., 2020). This point needs further research.

There is an accumulation of negative emotions in students, and this process seems to be independent of other factors. It is especially important to help students stay positive to avoid excessive feelings of stress. Individuals with low resilience need more timely attention and support. In addition, the relationship between loneliness and negative emotions suggests that social connections are more important than ever for individual mental health significance in an epidemic. However, the establishment of such relationships is also more difficult than ever, and how to maintain supportive social connections in an epidemic would be an interesting topic (Chu, 2022).

Raftopoulou et al. (2022) confirms that positive psychology interventions can significantly reduce negative emotions and enhance individual motivation and mental resilience. Self-compassion intervention can also help young people reduce anxiety and depression. It can even benefit them for a lifelong time (Athanasakou et al., 2020; Karakasidou et al., 2021a,b). During the epidemic, several new digital products were developed to combat loneliness or for psychotherapy (Shah et al., 2020). How to use provide useful positive psychological intervention is not only helpful for a population under epidemic conditions, but is also important for psychological services for workers in special environments such as oil fields, submarines, and polar regions.

In addition to external support, students can also cope with the negative effects of lockdowns by adjusting their own cognition. Student’s attitude to new method of learning can influences their emotional experience. If they can view online-learning as a kind of resource but not obstacle, they will feel less fear and depressed (Novara et al., 2022). Maintain life continuity is a kind of psychological resilience strategy. People’s attitudes toward time are related to their levels of distress anxiety and well-being. Finding connection with past experiences and future goals can help they overcoming the present constrain (Fortuna et al., 2021).

A limitation of the current research should be noted. The data in this paper are based on a survey of students during a winter break when school is strictly closed for epidemic prevention. For students in this study, they were living in a campus with relatively low population density. The density of the campus population will increase after the school year starts and students may feel less lonely, while at the same time the academic pressure will also increase accordingly. Because the school never be strictly closed for a long time in the following semester, the data of students in the higher campus density cannot be collected.

## Data availability statement

The original contributions presented in the study are included in the article/Supplementary material, further inquiries can be directed to the corresponding author.

## Ethics statement

The studies involving humans were approved by Institutional Review Board of the Faculty of Psychology, BNU. The studies were conducted in accordance with the local legislation and institutional requirements. The participants provided their written informed consent to participate in this study. Written informed consent was obtained from the individual(s) for the publication of any potentially identifiable images or data included in this article.

## Author contributions

QW made a major contribution for study carrying, data analysis and manuscript writing. GY participated in the topic select, study design, and helped to draft the manuscript. YH guided the data interpretation and gave a lot of valuable advice on the writing of manuscript. GD helped to draft the manuscript and participated in its revision. YL participated in the writing of manuscript. All authors contributed to the article and approved the submitted version.
