# Supplementary material for: Stress and emotion in a locked campus: the moderating effects of resilience and loneliness
Source: Front Psychol. 2024 Jan 8;14:1168020. doi: 10.3389/fpsyg.2023.1168020 (PMC10800410; doi:10.3389/fpsyg.2023.1168020)
Supplement: Supplementary file 1 [file Table_1.DOCX]

Mplus VERSION 8.3

MUTHEN & MUTHEN

04/11/2023 8:27 PM

INPUT INSTRUCTIONS

DATA: FILE = Two-Level Data.csv;

VARIABLE: NAMES ARE NE PE STR RE LON PERSON TIME;

USEVARIABLES NE PE STR RE LON;

Lagged = NE(1) ;

BETWEEN=RE LON;

cluster=PERSON;

TINTERVAL = time(1)

Define: Center RE LON (Grandmean);

ANALYSIS: TYPE = TWOLEVEL RANDOM;

estimator=bayes;

biter=(1000);

bseed=1028;

MODEL:

%WITHIN%

PHI | NE on NE&1;

! negative emotion is regressed on Lag-1 negative emotion, the slope is latent;

BETA | NE on STR;

! negative emotion is regressed on stress, the slope is latent;

ETA | NE on PE;

! negative emotion is regressed on positive emotion, the slope is latent;

NE; !within-level variance, sigma^2;

%BETWEEN%

[NE]; !mean intercept, gamma_00;

[PHI]; !mean of autoregressive slope,gamma_10;

[BETA]; !mean of TVC slope, gamma_20;

[STR];

[PE];

NE; !intercept variance, tau_00;

PHI; !autoregressive slope variance, tau_11;

BETA; !TVC slope variance, tau_22;

STR;

PE

NE on RE LON STR PE;

!resilience and loneliness predict the intercept,gamma_01 and gamma_02;

PHI on RE LON;

!resilience and loneliness predict the autoregressive slope,gamma_11 and gamma_12;

BETA on RE LON;

!resilience and loneliness predict the TVC slope,gamma_21 and gamma_22;

ETA on RE LON;

output: standardized;

*** WARNING in MODEL command

In the MODEL command, the x variable on the WITHIN level has been turned into a

y variable to enable latent variable decomposition. This variable will be treated

as a y-variable on both levels: PE

*** WARNING in MODEL command

In the MODEL command, the x variable on the WITHIN level has been turned into a

y variable to enable latent variable decomposition. This variable will be treated

as a y-variable on both levels: STR

*** WARNING

Data set contains cases with missing on x-variables converted to dependent variables.

The autocorrelation of these variables should be included in the model and the variables

added to the LAGGED option. The following converted x-variable(s) have cases with missing:

STR (2261 cases with missing data)

PE (2261 cases with missing data)

3 WARNING(S) FOUND IN THE INPUT INSTRUCTIONS

SUMMARY OF ANALYSIS

Number of groups 1

Number of observations 7239

Number of dependent variables 3

Number of independent variables 3

Number of continuous latent variables 3

Observed dependent variables

Continuous

NE PE STR

Observed independent variables

RE LON NE&1

Continuous latent variables

PHI BETA ETA

Variables with special functions

Cluster variable PERSON

Within variables

NE&1

Between variables

RE LON

Centering (GRANDMEAN)

RE LON

Estimator BAYES

Specifications for Bayesian Estimation

Point estimate MEDIAN

Number of Markov chain Monte Carlo (MCMC) chains 2

Random seed for the first chain 1028

Starting value information UNPERTURBED

Algorithm used for Markov chain Monte Carlo GIBBS(PX1)

Convergence criterion 0.500D-01

Maximum number of iterations 50000

K-th iteration used for thinning 1

Input data file(s)

Two-Level Data.csv

Input data format FREE

SUMMARY OF DATA

Number of clusters 88

Size (s) Cluster ID with Size s

54 84

67 86

81 78 71 76 88

82 53 54 65 67 32 33 35 79 36 85 40 44

83 19 20 21 22 23 24 25 26 27 28 29 30 31 1 2 34 3 4 37

38 39 5 41 42 43 6 45 46 47 48 49 50 51 52 7 8 55 56

57 58 59 60 61 62 63 64 9 66 10 68 69 70 11 72 73 74

75 12 77 13 14 80 81 82 83 15 16 17 87 18

COVARIANCE COVERAGE OF DATA

Minimum covariance coverage value 0.100

Number of missing data patterns 4

PROPORTION OF DATA PRESENT

Covariance Coverage

NE PE STR RE LON

________ ________ ________ ________ ________

NE 0.688

PE 0.688 0.688

STR 0.688 0.688 0.688

RE 0.688 0.688 0.688 1.000

LON 0.688 0.688 0.688 1.000 1.000

UNIVARIATE SAMPLE STATISTICS

UNIVARIATE HIGHER-ORDER MOMENT DESCRIPTIVE STATISTICS

Variable/ Mean/ Skewness/ Minimum/ % with Percentiles

Sample Size Variance Kurtosis Maximum Min/Max 20%/60% 40%/80% Median

NE 0.000 0.956 -1.124 12.35% -0.892 -0.543 -0.194

4978.000 1.000 0.347 3.523 0.10% 0.038 0.851

PE 0.000 0.234 -1.749 2.85% -0.932 -0.349 0.001

4978.000 1.000 -0.660 2.919 0.02% 0.235 0.935

STR 0.000 -0.069 -1.678 13.34% -0.835 0.008 0.008

4978.000 1.000 -0.824 1.693 10.93% 0.008 0.850

RE 0.000 -0.432 -2.766 1.14% -0.774 -0.276 0.001

88.000 0.973 0.322 2.103 1.14% 0.222 0.941

LON 0.000 0.087 -2.068 1.14% -0.973 -0.289 -0.106

88.000 0.979 -0.771 1.901 1.14% 0.259 0.897

WARNING: PROBLEMS OCCURRED IN SEVERAL ITERATIONS IN THE COMPUTATION OF THE STANDARDIZED ESTIMATES FOR SEVERAL

CLUSTERS. THIS IS MOST LIKELY DUE TO AR COEFFICIENTS GREATER THAN 1 OR PARAMETERS GIVING NON-STATIONARY MODELS.

SUCH POSTERIOR DRAWS ARE REMOVED. THE FOLLOWING CLUSTERS HAD SUCH PROBLEMS:

84

THE MODEL ESTIMATION TERMINATED NORMALLY

USE THE FBITERATIONS OPTION TO INCREASE THE NUMBER OF ITERATIONS BY A FACTOR

OF AT LEAST TWO TO CHECK CONVERGENCE AND THAT THE PSR VALUE DOES NOT INCREASE.

MODEL FIT INFORMATION

Number of Free Parameters 25

Information Criteria

Deviance (DIC) 45848.534

Estimated Number of Parameters (pD) 6395.589

MODEL RESULTS

Posterior One-Tailed 95% C.I.

Estimate S.D. P-Value Lower 2.5% Upper 2.5% Significance

Within Level

Variances

PE 0.388 0.008 0.000 0.373 0.404 *

STR 0.575 0.011 0.000 0.554 0.597 *

Residual Variances

NE 0.210 0.004 0.000 0.202 0.219 *

Between Level

PHI ON

RE -0.043 0.027 0.053 -0.094 0.010

LON 0.011 0.027 0.353 -0.041 0.063

BETA ON

RE -0.050 0.020 0.009 -0.089 -0.009 *

LON 0.012 0.019 0.278 -0.028 0.048

ETA ON

RE -0.012 0.028 0.347 -0.067 0.044

LON 0.037 0.028 0.087 -0.019 0.092

NE ON

RE -0.017 0.067 0.399 -0.153 0.119

LON 0.169 0.064 0.003 0.043 0.294 *

STR 0.812 0.101 0.000 0.609 1.006 *

PE 0.132 0.082 0.055 -0.033 0.293

Means

PE -0.016 0.088 0.438 -0.179 0.158

STR 0.009 0.074 0.445 -0.151 0.139

Intercepts

NE 0.005 0.060 0.464 -0.117 0.125

PHI 0.319 0.026 0.000 0.265 0.368 *

BETA 0.281 0.020 0.000 0.242 0.321 *

ETA -0.088 0.027 0.000 -0.139 -0.032 *

Variances

PE 0.645 0.104 0.000 0.493 0.892 *

STR 0.425 0.070 0.000 0.317 0.586 *

Residual Variances

NE 0.311 0.055 0.000 0.225 0.444 *

PHI 0.039 0.009 0.000 0.025 0.059 *

BETA 0.023 0.005 0.000 0.015 0.035 *

ETA 0.048 0.010 0.000 0.032 0.072 *

STANDARDIZED MODEL RESULTS

STDYX Standardization

Posterior One-Tailed 95% C.I.

Estimate S.D. P-Value Lower 2.5% Upper 2.5% Significance

Within-Level Standardized Estimates Averaged Over Clusters

PHI | NE ON

NE&1 0.319 0.018 0.000 0.283 0.349 *

BETA | NE ON

STR 0.352 0.014 0.000 0.325 0.378 *

ETA | NE ON

PE -0.092 0.014 0.000 -0.120 -0.065 *

Variances

PE 1.000 0.000 0.000 1.000 1.000

STR 1.000 0.000 0.000 1.000 1.000

Residual Variances

NE 0.648 0.012 0.000 0.626 0.671 *

Between Level

PHI ON

RE -0.149 0.091 0.053 -0.319 0.035

LON 0.038 0.092 0.353 -0.140 0.214

BETA ON

RE -0.224 0.087 0.009 -0.386 -0.042 *

LON 0.054 0.085 0.278 -0.121 0.212

ETA ON

RE -0.037 0.085 0.347 -0.197 0.126

LON 0.119 0.086 0.087 -0.059 0.283

NE ON

RE -0.015 0.059 0.399 -0.132 0.099

LON 0.150 0.058 0.003 0.038 0.270 *

STR 0.672 0.064 0.000 0.527 0.777 *

PE 0.132 0.081 0.055 -0.037 0.293

Means

PE -0.019 0.108 0.438 -0.219 0.198

STR 0.014 0.111 0.445 -0.223 0.208

Intercepts

NE 0.006 0.076 0.464 -0.142 0.162

PHI 1.598 0.234 0.000 1.157 2.070 *

BETA 1.796 0.223 0.000 1.372 2.268 *

ETA -0.390 0.128 0.000 -0.630 -0.145 *

Variances

PE 1.000 0.000 0.000 1.000 1.000

STR 1.000 0.000 0.000 1.000 1.000

Residual Variances

NE 0.494 0.080 0.000 0.353 0.663 *

PHI 0.965 0.029 0.000 0.886 0.998 *

BETA 0.940 0.040 0.000 0.837 0.991 *

ETA 0.976 0.024 0.000 0.909 0.999 *

STDY Standardization

Posterior One-Tailed 95% C.I.

Estimate S.D. P-Value Lower 2.5% Upper 2.5% Significance

Within-Level Standardized Estimates Averaged Over Clusters

PHI | NE ON

NE&1 0.528 0.032 0.000 0.465 0.581 *

BETA | NE ON

STR 0.352 0.014 0.000 0.325 0.378 *

ETA | NE ON

PE -0.092 0.014 0.000 -0.120 -0.065 *

Variances

PE 1.000 0.000 0.000 1.000 1.000

STR 1.000 0.000 0.000 1.000 1.000

Residual Variances

NE 0.648 0.012 0.000 0.626 0.671 *

Between Level

PHI ON

RE -0.212 0.130 0.053 -0.456 0.050

LON 0.055 0.131 0.353 -0.199 0.305

BETA ON

RE -0.320 0.125 0.009 -0.552 -0.061 *

LON 0.077 0.122 0.278 -0.173 0.303

ETA ON

RE -0.053 0.122 0.347 -0.281 0.180

LON 0.169 0.123 0.087 -0.084 0.404

NE ON

RE -0.021 0.084 0.399 -0.188 0.141

LON 0.214 0.082 0.003 0.053 0.385 *

STR 0.672 0.064 0.000 0.527 0.777 *

PE 0.132 0.081 0.055 -0.037 0.293

Means

PE -0.019 0.108 0.438 -0.219 0.198

STR 0.014 0.111 0.445 -0.223 0.208

Intercepts

NE 0.006 0.076 0.464 -0.142 0.162

PHI 1.598 0.234 0.000 1.157 2.070 *

BETA 1.796 0.223 0.000 1.372 2.268 *

ETA -0.390 0.128 0.000 -0.630 -0.145 *

Variances

PE 1.000 0.000 0.000 1.000 1.000

STR 1.000 0.000 0.000 1.000 1.000

Residual Variances

NE 0.494 0.080 0.000 0.353 0.663 *

PHI 0.965 0.029 0.000 0.886 0.998 *

BETA 0.940 0.040 0.000 0.837 0.991 *

ETA 0.976 0.024 0.000 0.909 0.999 *

STD Standardization

Posterior One-Tailed 95% C.I.

Estimate S.D. P-Value Lower 2.5% Upper 2.5% Significance

Within-Level Standardized Estimates Averaged Over Clusters

PHI | NE ON

NE&1 0.319 0.018 0.000 0.283 0.349 *

BETA | NE ON

STR 0.280 0.011 0.000 0.259 0.302 *

ETA | NE ON

PE -0.087 0.014 0.000 -0.116 -0.061 *

Variances

PE 0.387 0.009 0.000 0.372 0.404 *

STR 0.575 0.012 0.000 0.554 0.599 *

Residual Variances

NE 0.210 0.011 0.000 0.201 0.219 *

Between Level

PHI ON

RE -0.212 0.130 0.053 -0.456 0.050

LON 0.055 0.131 0.353 -0.199 0.305

BETA ON

RE -0.320 0.125 0.009 -0.552 -0.061 *

LON 0.077 0.122 0.278 -0.173 0.303

ETA ON

RE -0.053 0.122 0.347 -0.281 0.180

LON 0.169 0.123 0.087 -0.084 0.404

NE ON

RE -0.017 0.067 0.399 -0.153 0.119

LON 0.169 0.064 0.003 0.043 0.294 *

STR 0.812 0.101 0.000 0.609 1.006 *

PE 0.132 0.082 0.055 -0.033 0.293

Means

PE -0.016 0.088 0.438 -0.179 0.158

STR 0.009 0.074 0.445 -0.151 0.139

Intercepts

NE 0.005 0.060 0.464 -0.117 0.125

PHI 1.598 0.234 0.000 1.157 2.070 *

BETA 1.796 0.223 0.000 1.372 2.268 *

ETA -0.390 0.128 0.000 -0.630 -0.145 *

Variances

PE 0.645 0.104 0.000 0.493 0.892 *

STR 0.425 0.070 0.000 0.317 0.586 *

Residual Variances

NE 0.311 0.055 0.000 0.225 0.444 *

PHI 0.965 0.029 0.000 0.886 0.998 *

BETA 0.940 0.040 0.000 0.837 0.991 *

ETA 0.976 0.024 0.000 0.909 0.999 *

R-SQUARE

Within-Level R-Square Averaged Across Clusters

Posterior One-Tailed 95% C.I.

Variable Estimate S.D. P-Value Lower 2.5% Upper 2.5%

NE 0.352 0.012 0.000 0.329 0.374

Between Level

Posterior One-Tailed 95% C.I.

Variable Estimate S.D. P-Value Lower 2.5% Upper 2.5%

NE 0.506 0.080 0.000 0.337 0.646

Posterior One-Tailed 95% C.I.

Variable Estimate S.D. P-Value Lower 2.5% Upper 2.5%

PHI 0.035 0.029 0.000 0.002 0.114

BETA 0.060 0.040 0.000 0.009 0.160

ETA 0.024 0.024 0.000 0.001 0.087

DIAGRAM INFORMATION

Mplus diagrams are currently not available for multilevel analysis.

No diagram output was produced.

Beginning Time: 20:27:39

Ending Time: 20:27:58

Elapsed Time: 00:00:19

MUTHEN & MUTHEN

3463 Stoner Ave.

Los Angeles, CA 90066

Tel: (310) 391-9971

Fax: (310) 391-8971

Web: www.StatModel.com

Support: Support@StatModel.com

Copyright (c) 1998-2019 Muthen & Muthen
